# Antidepressant-Warfarin Interaction and Associated Gastrointestinal Bleeding Risk in a Case-Control Study

**DOI:** 10.1371/journal.pone.0021447

**Published:** 2011-06-24

**Authors:** Hedi Schelleman, Colleen M. Brensinger, Warren B. Bilker, Sean Hennessy

**Affiliations:** 1 Center for Clinical Epidemiology and Biostatistics, Department of Biostatistics and Epidemiology, University of Pennsylvania School of Medicine, Philadelphia, Pennsylvania, United States of America; 2 Center for Education and Research on Therapeutics, University of Pennsylvania School of Medicine, Philadelphia, Pennsylvania, United States of America; Federal University of Rio de Janeiro, Brazil

## Abstract

**Background:**

Bleeding is the most common and worrisome adverse effect of warfarin therapy. One of the factors that might increase bleeding risk is initiation of interacting drugs that potentiate warfarin. We sought to evaluate whether initiation of an antidepressant increases the risk of hospitalization for gastrointestinal bleeding in warfarin users.

**Methodology/Principal Findings:**

Medicaid claims data (1999–2005) were used to perform an observational case-control study nested within person-time exposed to warfarin in those ≥18 years. In total, 430,455 warfarin users contributed 407,370 person-years of warfarin use. The incidence rate of hospitalization for GI bleeding among warfarin users was 4.48 per 100 person-years (95% CI, 4.42–4.55). Each gastrointestinal bleeding cases was matched to 50 controls based on index date and state. Warfarin users had an increased odds ratio of gastrointestinal bleeding upon initiation of citalopram (OR = 1.73 [95% CI, 1.25–2.38]), fluoxetine (OR = 1.63 [95% CI, 1.11–2.38]), paroxetine (OR = 1.64 [95% CI, 1.27–2.12]), amitriptyline (OR = 1.47 [95% CI, 1.02–2.11]). Also mirtazapine, which is not believed to interact with warfarin, increased the risk of GI bleeding (OR = 1.75 [95% CI, 1.30–2.35]).

**Conclusions/Significance:**

Warfarin users who initiated citalopram, fluoxetine, paroxetine, amitriptyline, or mirtazapine had an increased risk of hospitalization for gastrointestinal bleeding. However, the elevated risk with mirtazapine suggests that a drug-drug interaction may not have been responsible for all of the observed increased risk.

## Introduction

Warfarin is highly effective in reducing the risk of thromboembolic events. With an estimated two million people initiating warfarin therapy each year in the US [1], it is one of the top 20 drugs prescribed in the US. Bleeding is the most common and worrisome adverse effect of warfarin therapy, with an annual incidence of major bleeding of 4–8% [2], [3]. One of the factors that might increase bleeding risk is initiation of interacting drugs that potentiate warfarin.

Depression often coexists with cardiovascular disease, and nearly 7% of warfarin users are co-prescribed antidepressants [4]. Commonly used drug-drug interaction compendia in the US warn about potential interactions between warfarin and several selective serotonin reuptake inhibitors (SSRIs), serotonin-norepinephrine reuptake inhibitors (SNRIs), and tricyclic antidepressants (TCAs) [5]–[7]. These agents might increase bleeding risk by inhibiting CYP2C9 (mainly fluoxetine and fluvoxamine [8]), which metabolizes the more potent (S)-enantiomer of warfarin; by inhibiting CYP1A2 or CYP3A4, which metabolizes the less potent (R)-enantiomer of warfarin; and/or by blocking serotonin reuptake by platelets, which may result in impaired platelet aggregation (mainly SSRIs [9]–[15]).

Three observational studies have evaluated the risk of bleeding during exposure to antidepressants in warfarin and coumarin users [4], [16], [17]. However, their results have conflicted. In two studies, a statistically significantly increased bleeding risk was found [16], [17], while in a third no increased bleeding risk was found [4]. Further, in all of the studies, investigators assumed that bleeding risk was constant after initiation of the antidepressant. In contrast, we would expect that the GI bleeding risk in warfarin users would be the highest shortly after initiation of an antidepressant, and would decline subsequently because of depletion of susceptibles (i.e., patients who are susceptible to bleeding from the drug-drug interaction develop the event early) [18]. Therefore, we sought to evaluate whether warfarin users who initiated antidepressants had a higher risk of GI bleeding shortly after antidepressant initiation.

## Methods

### Settings and design

This observational case-control study used pre-existing Medicaid data of California, Florida, New York, Ohio, and Pennsylvania from 1999 to 2005. In total, these five states include approximately 13 million Medicaid enrollees, which account for about 35% of the Medicaid population. The data were obtained from the Centers for Medicare and Medicaid Services (CMS) [19], as were linked Medicare data on all Medicare-Medicaid dual eligibles. This data source contains health care claims for hospital, medical, and outpatient pharmaceutical coverage linked to enrollment information. A series of quality assurance analyses found low rates of abnormalities in this data source, suggesting that the data are of high quality [20].

This study was approved by the University of Pennsylvania's Committee on Studies Involving Human Beings, which granted waivers of informed consent and Health Insurance Portability and Accountability Act authorization.

### Eligible subjects and person-time in this case-control study

Only person-time exposed to warfarin (outpatient prescriptions only) in those aged 18 years and older between January 1, 1999 and December 1, 2005 was included. We assumed that the duration of a warfarin prescription was equivalent to the number of tablets dispensed with a maximum duration of 30 days. Prescriptions that were filled on the same day were combined. We used a maximum of 30 days because prescriptions covered by Medicaid in the five study states are typically dispensed in 30-day increments. This assumption was confirmed by examining the frequency distribution of the number of pills dispensed and the number of days between subsequent warfarin prescriptions for each warfarin user. Nonetheless, we also performed a sensitivity analyses assuming that each warfarin prescription was equivalent to twice the number of tablets dispensed with maximum duration of 60 days. The rationale for this sensitivity analyses was to allow for non-adherence, and include events that may have occurred shortly after cessation of therapy.

The observation period ended with either a hospitalization for GI bleeding, the presumed end of the last warfarin prescription, discontinuation of Medicaid eligibility, or end of the study period, whichever occurred first. Further, we excluded all warfarin users who filled a prescription for an antidepressant of interest 90 days before or on the same days as their first observed warfarin prescription, because the goal of this study was to evaluate the safety of initiation of SSRIs, SNRIs, and TCAs in patients already receiving warfarin. In a secondary analysis, we evaluated whether duration of warfarin use influenced the ORs of interest. The rationale is that chronic warfarin users, defined as those with ≥three warfarin prescriptions on the index date, were expected to have been on a stable warfarin dose and have less frequent International Normalized Ratio (INR) measurements, and therefore to be more likely to experience a bleeding complication due to a drug-drug interaction compared to a new warfarin initiator. In addition, because re-initiators might have a different GI bleeding risk than continuing warfarin users, we conducted a sensitivity analysis in which we excluded follow-up time after ≥180 days between consecutive warfarin prescriptions.

### Identification of cases and controls

Cases consisted of all individuals age 18 and above hospitalized with a principal or non-principal International Classification of Diseases, 9^th^ Revision (ICD-9) code for GI bleeding during eligible person-time exposed to warfarin. ICD-9 codes used to identify gastrointestinal bleeding events were: ulcer of esophagus with hemorrhage (530.21), gastric ulcer with hemorrhage (531.1*, 531.2*, 531.4*, 531.6*), duodenal ulcer with hemorrhage (532.1*, 532.2*, 532.4*, 532.6*), peptic ulcer with hemorrhage (533.1*, 533.2*, 533.4*, 533.6*), gastrojejunal ulcer with hemorrhage (534.1*, 534.2*, 534.4*, 534.6*), gastritis and duodenitis with hemorrhage (535.01, 535.11, 535.21, 535.31, 535.41, 535.51, 535.61), angiodysplasia or dieulafoy lesion of stomach and duodenum with hemorrhage (537.83, 537.84), diverticul of intestine with hemorrhage (562.02, 562.03, 562.12, 562.13), angiodysplasia or dieulafoy lesion of intestine with hemorrhage (569.85, 569.86), and gastrointestinal hemorrhage (578.*). These codes have been shown to have a positive predictive value of 81% [21]. The index date for a case was the hospital admission date.

Fifty controls were selected at random for each case during eligible person-time exposed to warfarin, matched on index date and state, using incidence density sampling [22]. Eligible controls consisted of warfarin users who had not been hospitalized with a diagnosis code for GI bleeding by the day of the hospitalization of the case. The index date assigned to each control was the index date of their matched case.

### Co-exposure to an antidepressant

SSRIs, SNRIs, and TCAs that were listed as potentially interacting agents with warfarin in commonly used US drug interaction compendia (Drug Facts [5], MicroMedex 2.0 [6], or Drug Interactions Analysis and Management 2010 [7]) were considered as study agents of interest. To evaluate whether there might be residual confounding, mirtazapine was chosen as a reference drug. Mirtazapine has, as far as we know, not been suggested to interact with warfarin. We considered a warfarin user co-exposed to antidepressant of interest if a prescription was filled 29 days prior or on the index-date. The rationale for this was that the average duration of an antidepressant prescription was 30 days. Warfarin users exposed to two or more different antidepressants in the index date were excluded.

Each case and control exposed to one antidepressant on the index date was classified into one of three categories based on number of days between the index date and their first observed antidepressant prescription, i.e., 0–29 (first antidepressant prescription), 30–59 (second antidepressant prescription), 60–119 days (third and fourth antidepressant prescription). After a gap of ≥180 days between consecutive antidepressant prescriptions, the numbering was restarted. In a sensitivity analysis, we excluded follow-up time after a ≥180 day gap between consecutive prescriptions. To avoid having an insufficient number of events in multivariable models, we did not evaluate antidepressants with fewer than eleven exposed cases or controls for any of the time categories.

### Ascertainment of potential confounding variables

The appendix lists all of the potential confounding variables that were ascertained. The following four classes of potential confounding factors were defined as of the index date: 1) demographic factors; 2) chronic diseases, defined as diagnosis ever before the index date; 3) use of agents that could increase or decrease the bleeding risk; and 4) use of agents that could potentially interact with warfarin as defined by MicroMedex [6], defined as a prescription in the prior 30 days.

### Statistical analysis

We first measured the incidence rate of GI bleeding in the cohort of warfarin users. Next, we calculated the odds ratios (ORs) and 95% confidence intervals (CI) for the association between initiation of an antidepressant and hospital admission for GI bleeding using conditional logistic regression. In the minimally adjusted model, we adjusted for age, gender, race, and number of prior warfarin prescriptions (classified as 0, 1, 2, or 3+). We then examined each potential confounding factor individually, and, if the factor changed any of the ORs of interest by ≥5%, it was considered a confounder and retained in the fully adjusted model [23]. To determine whether this confounder selection approach might have missed measured confounders, we compared the results of the fully adjusted model with a saturated model that included all potential confounders.

## Results

In total, 430,455 warfarin users contributed 407,370 person-years of warfarin use. The incidence rate of hospitalization for GI bleeding was 4.48 per 100 person-years (95% CI, 4.42–4.55). After the exclusion of 110,600 warfarin users (26%) who had filled a prescription for an antidepressant 0–89 days prior to their first warfarin prescription and 121 cases (0.9%) and 3369 controls (0.5%) who were exposed to two or more antidepressants on the index date, 13,026 cases of GI bleeding and 653,209 controls remained in the analysis. Table 1 presents the baseline characteristics of cases and controls.

**Table 1:** Characteristics on the index date of cases of gastrointestinal bleeding and controls exposed to warfarin.

Variables	Cases		Controls				
	N = 13,026		N = 653,209		Matched	Lower	Upper
	N	%	N	%	OR*	95% CI	95% CI
Age							
18–50	849	6.52	85,456	13.08	Reference		
51–60	1,208	9.27	79,866	12.23	1.53	1.40	1.67
60–70	2,336	17.93	123,848	18.96	1.91	1.76	2.06
70–80	3,901	29.95	179,088	27.42	2.21	2.05	2.38
81+	4,732	36.33	184,951	28.31	2.61	2.43	2.81
Gender, male	4,472	34.33	238,512	36.51	0.91	0.88	0.94
Race							
Caucasian	7,423	56.99	388,804	59.52	Reference		
African American	2,305	17.70	94,479	14.46	1.28	1.22	1.35
Other/Unknown	3,298	25.32	169,926	26.01	1.02	0.97	1.06
Nursing home resident	3,742	28.73	121,666	18.63	1.96	1.88	2.04
Dementia†	3,757	28.84	127,462	19.51	1.71	1.65	1.78
Renal disease†	3,893	29.89	94,605	14.48	2.55	2.46	2.65
Liver disease†	2,827	21.70	90,035	13.78	1.77	1.69	1.84
Prior GI bleeding‡	4,641	35.63	110,158	16.86	2.82	2.72	2.93
Use of acetaminophen§	3,189	24.48	113,315	17.35	1.55	1.49	1.61
Use of NSAID§	721	5.54	22,101	3.38	1.68	1.55	1.81
Use of levofloxacin§	818	6.28	18,097	2.77	2.36	2.20	2.54
Use of PPI§	3,300	25.33	123,180	18.86	1.48	1.42	1.54
Number of prior							
warfarin prescriptions							
0	1,537	11.80	28,539	4.37	3.17	3.00	3.35
1	957	7.35	28,350	4.34	2.00	1.87	2.14
2	746	5.73	28,846	4.42	1.54	1.43	1.66
3+	9,786	75.13	567,474	86.87	Reference		
Citalopram§	162	1.24	7,314	1.12	1.11	0.95	1.30
Escitalopram§	146	1.12	7,109	1.09	1.03	0.87	1.22
Fluoxetine§	114	0.88	5,490	0.84	1.04	0.87	1.26
Paroxetine§	258	1.98	11,932	1.83	1.09	0.96	1.23
Sertraline§	316	2.43	13,850	2.12	1.15	1.03	1.29
Venlafaxine§	77	0.59	4,133	0.63	0.94	0.75	1.17
Mirtazapine§	152	1.17	6,538	1.00	1.17	1.00	1.38
Amitriptyline§	122	0.94	5,001	0.77	1.22	1.02	1.47
Doxepin§	22	0.17	531	0.08	2.10	1.37	3.21
Nortriptyline§	20	0.15	1,147	0.18	0.88	0.56	1.36

CI = confidence interval; GI = gastrointestinal; OR = odds ratio; NSAID = non-steroidal anti-inflammatory drug; PPI = proton pump inhibitor.

*Adjusted for matching variables, i.e., index date and state.

† Ever in the past.

‡ Either an outpatient diagnosis for GI bleeding during warfarin therapy or a hospital admission for GI bleeding before initiating warfarin therapy, excluding the day prior to and the index date.

§ Prescription dispensed 0–29 days prior to the index date.

The minimally and fully adjusted ORs for the association between initiation of an antidepressant and GI bleeding for each of the three exposure categories of interest (first, second, or third or fourth antidepressant prescription) are presented in Table 2 and 3. During the *a priori* defined primary duration category for a drug-drug interaction (i.e., first antidepressant prescription) the minimally adjusted ORs ranged from 1.51 to 2.49 and were all statistically significant except for nortriptyline (Table 2). After adjusting for confounders that changed any of the ORs of interest, all ORs were attenuated and ranged from 1.18–1.75 (Table 3). The adjusted association between GI bleeding and initiating of citalopram (OR = 1.73), fluoxetine (OR = 1.63), paroxetine (OR = 1.64), amitriptyline (OR = 1.47), and the reference agent mirtazapine (OR = 1.75) remained statistically elevated. In fully adjusted analyses of the second and third or fourth prescription, no statistically significant associations were observed, except for amitriptyline during the third or fourth prescription (OR = 1.61). The results of the fully adjusted model were similar to the saturated model (data not shown). Extending the maximum duration of a warfarin prescription to 60 days, excluding warfarin users with a gap of ≥180 days between consecutive warfarin prescriptions, and antidepressant users with a gap of ≥180 days between consecutive antidepressant prescriptions gave similar results as those shown in Table 3 (data not shown).

**Table 2:** Minimally adjusted association between initiation of an antidepressant agent (exposed versus unexposed) and hospitalization for gastrointestinal bleeding in warfarin users.

Variables	0 to 29 days			30 to 59 days			60 to 119 days		
	(1^st^ prescription)*			(2^nd^ prescription)*			(3^rd^ or 4^th^ prescription)*		
	OR	Lower	Upper	OR	Lower	Upper	OR	Lower	Upper
		95% CI	95% CI		95% CI	95% CI		95% CI	95% CI
Citalopram	2.28	1.66	3.13	1.31	0.80	2.16	1.58	1.07	2.34
Escitalopram	1.58	1.10	2.27	1.31	0.82	2.10	1.27	0.86	1.88
Fluoxetine	1.94	1.34	2.83	1.06	0.55	2.06	0.84	0.45	1.56
Paroxetine	1.94	1.51	2.49	1.49	1.03	2.14	1.30	0.93	1.81
Sertraline	1.51	1.15	1.98	1.49	1.05	2.11	1.48	1.10	1.98
Venlafaxine	1.82	1.13	2.93	0.77	0.32	1.86	1.80	1.09	2.97
Amitriptyline	1.66	1.16	2.39	0.98	0.51	1.90	1.84	1.18	2.88
Nortriptyline	1.66	0.78	3.54	No data†			No data†		
Mirtazapine	2.49	1.86	3.33	0.92	0.52	1.63	1.34	0.90	2.00

*Adjusted for age, gender, race, and number of prior warfarin prescriptions filled on the index date.

† Insufficient number of exposed cases or controls to analyze.

**Table 3:** Fully adjusted association between initiation of an antidepressant agent (exposed versus unexposed) and hospitalization for gastrointestinal bleeding in warfarin users.

Variables	0 to 29 days			30 to 59 days			60 to 119 days		
	(1^st^ prescription)*			(2^nd^ prescription)*			(3^rd^ or 4^th^ prescription)*		
	OR	Lower	Upper	OR	Lower	Upper	OR	Lower	Upper
		95% CI	95% CI		95% CI	95% CI		95% CI	95% CI
Citalopram	1.73	1.25	2.38	1.05	0.63	1.73	1.24	0.84	1.85
Escitalopram	1.19	0.82	1.71	1.01	0.63	1.63	0.95	0.64	1.41
Fluoxetine	1.63	1.11	2.38	0.80	0.41	1.57	0.72	0.38	1.35
Paroxetine	1.64	1.27	2.12	1.29	0.90	1.87	1.07	0.77	1.49
Sertraline	1.18	0.90	1.56	1.23	0.87	1.75	1.19	0.89	1.60
Venlafaxine	1.43	0.88	2.31	0.59	0.24	1.45	1.31	0.79	2.17
Amitriptyline	1.47	1.02	2.11	0.82	0.42	1.59	1.61	1.03	2.53
Nortriptyline	1.45	0.68	3.12	No data†			No data†		
Mirtazapine	1.75	1.30	2.35	0.64	0.36	1.13	0.95	0.63	1.42

*Adjusted for age, gender, race, number of prior warfarin prescriptions filled on the index date, nursing home, use of dementia, liver disease, prior gastrointestinal bleed, renal disease, use of acetaminophen, use of levofloxacin, and proton pump inhibitors.

† Insufficient number of exposed cases or controls to analyze.

Exclusion of warfarin users who were nursing home residents resulted in statistically significant elevated odds immediately after escitalopram initiation (first prescription OR = 1.60, 95% CI: 1.01–2.55; Table 4).

**Table 4:** Association between initiation of an antidepressant agent (exposed versus unexposed) and hospitalization for gastrointestinal bleeding in warfarin users, after exclusion of nursing home residents.

Variables	0 to 29 days			30 to 59 days			60 to 119 days		
	(1^st^ prescription)*			(2^nd^ prescription)*			(3^rd^ or 4^th^ prescription)*		
	OR	Lower	Upper	OR	Lower	Upper	OR	Lower	Upper
		95% CI	95% CI		95% CI	95% CI		95% CI	95% CI
Citalopram	1.83	1.18	2.86	1.09	0.51	2.31	1.52	0.91	2.56
Escitalopram	1.60	1.01	2.55	1.36	0.70	2.64	0.95	0.51	1.78
Fluoxetine	1.88	1.16	3.07	0.68	0.25	1.83	0.87	0.39	1.94
Paroxetine	1.58	1.14	2.19	1.68	1.10	2.55	1.29	0.86	1.95
Sertraline	1.37	0.98	1.94	1.51	0.98	2.34	1.41	0.96	2.07
Venlafaxine	1.48	0.78	2.81	0.59	0.15	2.39	2.01	1.09	3.69
Amitriptyline	1.50	1.01	2.23	0.88	0.43	1.77	1.77	1.10	2.84
Nortriptyline	1.81	0.84	3.87	No data†			No data†		
Mirtazapine	2.57	1.60	4.11	0.63	0.20	1.99	1.34	0.63	2.85

*Adjusted for age, gender, race, number of prior warfarin prescriptions filled on the index date, nursing home, use of dementia, liver disease, prior gastrointestinal bleed, renal disease, use of acetaminophen, use of levofloxacin, and proton pump inhibitors.

† Insufficient number of exposed cases or controls to analyze.

Initiation of antidepressants resulted in slightly higher fully-adjusted ORs for the first antidepressant prescription in chronic warfarin users compared to all warfarin users (citalopram: OR = 1.98, 95% CI: 1.37–2.88; escitalopram: OR = 1.32, 95% CI: 0.87–2.00; fluoxetine: OR = 1.98, 95% CI: 1.29–3.04; paroxetine: OR = 1.53, 95% CI: 1.10–2.13, sertraline: OR = 1.28, 95% CI: 0.90–1.81; venlafaxine: OR = 1.37, 95% CI: 0.74–2.54; amitriptyline: OR = 2.00, 95% CI: 1.35–2.97; nortriptyline: OR = 1.48, 95% CI: 0.65–3.40; mirtazapine: OR = 2.07, 95% CI: 1.47–2.92).

## Discussion

We found that warfarin users who initiated citalopram, fluoxetine, paroxetine, amitriptyline, or mirtazapine had approximately a 1.5-fold odds of serious GI bleeding during their first antidepressant prescription compared with warfarin users who did not fill a prescription for an antidepressant. Further, we found, as expected, that the risk of GI bleeding in warfarin users generally appeared to decline after the first antidepressant prescription period. However, the elevated odds ratio for mirtazapine, which is not believed to interact with warfarin, suggests that a drug-drug interaction may not have been responsible for all of the observed increased risk.

Three prior studies have evaluated the risk of GI bleeding in warfarin users co-exposed to antidepressants. In the first case-control study, use of antidepressants in the past 42 days could not be studied because of small number of co-exposed cases, and use in the past 90 days was not associated with hospitalization for upper GI bleeding in warfarin users (fluoxetine/fluvoxamine OR = 1.2, 95% CI: 0.8–1.7; other SSRIs OR = 1.1, 95% CI: 0.9–1.4; secondary TCAs OR = 0.7, 95% CI: 0.4–1.4) [4]. Thus while this study could not examine an early rise in risk with antidepressant initiation, results are consistent with no increased risk after the first antidepressant prescription. In a second case-control study, coumarin users hospitalized for GI bleeding had no statistically significant odds (OR = 0.8, 95% CI: 0.4–1.5) of being co-exposed to SSRIs in the past 30 days [16]. However, this study assumed that the risk after initiating an SSRI remains constant, and therefore would have likely have missed an initial increased risk if present. In a small cohort study, warfarin users co-exposed to SSRIs had a 3.49-fold (95% CI: 1.37–8.91) hazard of hospitalization for bleeding, but this study adjusted for few clinical variables [16].

While some studies have shown that SSRIs themselves are associated with bleeding even without warfarin [9], [11], [12], [14], not all studies have found this [10], [15]. In our study, several of the SSRIs were associated with GI bleeding, but there did not seem to be a relationship with affinitity for the serotonin reuptake transporter (paroxetine>sertraline>fluoxetine>citalopram [24]). Therefore, our data do not support the hypothesis that blocking of serotonin reuptake of platelets is responsible for the observed associations.

Our study had several limitations. The main limitation was the potential for residual confounding by unmeasured factors, such as depression and its severity, smoking, alcohol use, body mass area, and/or use of over the counter medications. Therefore, it remains questionable whether the observed increased bleeding risk during the first antidepressant prescription is due to a drug-drug interaction or the result unmeasured confounding (i.e., warfarin users with depression might have at higher risk than non-depressed warfarin users). However, during the third or fourth antidepressant prescription most of the ORs were close to 1, suggesting either that confounding by unmeasured factors did not have a major effect, depletion of susceptibles, or that depression treatment was associated with a reduced risk. Another limitation is the relative limited number of individuals that initiated antidepressant therapy, which resulted in wide 95% CI and limited our ability to study all drugs and all exposure windows of interest. Further, for some of the agents, the point estimates were elevated without statistically significance.

In conclusion, our finding suggest that there is an increased risk of GI bleeding after initiation of citalopram, fluoxetine, paroxetine, amitriptyline, and mirtazapine, and possible venlafaxine and nortriptyline. However, it remains debatable whether the observed increased bleeding risk during the first antidepressant prescription is due to a drug-drug interaction or the characteristics of patients receiving these agents. Regardless of mechanisms, increased clinical vigilance is prudent for patients on warfarin who initiate an antidepressant drug.
