# Correction: Antidepressant-Warfarin Interaction and Associated Gastrointestinal Bleeding Risk in a Case-Control Study

**DOI:** 10.1371/journal.pone.0121926

**Published:** 2015-03-26

**Authors:** The PLOS ONE Staff

There are errors in the second sentence of the “Identification of cases and controls” subsection of the Methods. The correct sentence is: ICD-9 codes used to identify gastrointestinal bleeding events were: ulcer of esophagus with hemorrhage (530.21), gastric ulcer with hemorrhage (531.0*, 531.2*, 531.4*, 531.6*), duodenal ulcer with hemorrhage (532.0*, 532.2*, 532.4*, 532.6*), peptic ulcer with hemorrhage (533.0*, 533.2*, 533.4*, 533.6*), gastrojejunal ulcer with hemorrhage (534.0*, 534.2*, 534.4*, 534.6*), gastritis and duodenitis with hemorrhage (535.01, 535.11, 535.21, 535.31, 535.41, 535.51, 535.61), angiodysplasia or dieulafoy lesion of stomach and duodenum with hemorrhage (537.83, 537.84), diverticul of intestine with hemorrhage (562.02, 562.03, 562.12, 562.13), angiodysplasia or dieulafoy lesion of intestine with hemorrhage (569.85, 569.86), and gastrointestinal hemorrhage (578.*).
